# Extension of Preliminary Education *Versus* Extension of the Dental Course

**Published:** 1905-09

**Authors:** R. H. Hofheinz

**Affiliations:** Rochester, N. Y.


### International Dental Journal.

Vol. XXVI. September, 1905. No. 9.

### Original Communications.[1]

### EXTENSION OF PRELIMINARY EDUCATION *VERSUS* EXTENSION OF THE DENTAL COURSE.[2]

BY R. H. HOFHEINZ, D.D.S.,[3] ROCHESTER, N. Y.

A great deal of agitation has recently been exhibited by all men concerned with the education of dental students. Some acrimonious feeling has emanated from the different aspects of the question, and some schools have directly been accused of insisting on returning to a three years' course, because the life and income of the school depended on the brevity of the course. As in all questions of any kind, much has been said and done in the right direction, and much has been said and done that would better have remained unsaid and undone.

It would be carrying water to the ocean should I speak of the gradual developments of the curriculum at our dental schools; they are a matter of history, and form one of the proudest parts of American University development. In this age of keenest competition, there is in every calling and profession a well-founded

[1] The editor and publishers are not responsible for the views of authors of papers published in this department, nor for any claim to novelty, or otherwise, that may be made by them. No papers will be received for this department that have appeared in any other journal published in the country.

[2] Read at the banquet given upon the tenth anniversary of the So ciety, April 25, 1905.

[3] Professor Operative Dentistry, University of Buffalo.

endeavor to raise the professional standard to the highest possible degree. Sense of honor and personal interest of the honest worker, as well as regard for the public welfare, demand it. The inferior work of the poorly educated endangers, always and everywhere, the material and moral welfare of the community. It prevents the public from attaining the best possible products of labor and the highest accomplishments of the pursuit of science; it causes waste of money on work of inferior value and often jeopardizes life and health, by subjecting them to unskilled hands and poorly trained minds. It fosters insincerity and fraud in competing with thoroughly trained and educated workers, by claiming ability to secure results only obtainable by long experience, close study, and application of the well-trained individual.

These facts create an ever-increasing tendency to suppress the quack in every profession, by establishing, whenever possible, a standard of skill and knowledge as a *conditio sine qua non* for admission to practice. Such a standard can only be established by members of the profession concerned, and from the consciousness of this fact arose the tendency to unite for the defence and preservation of common interests. Schools for the proper training of the dental students were established, and thus opportunities were offered for that high degree of special education which has distinguished so many members of our profession.

About fifty years ago the dental colleges had a curriculum consisting of a two years' course, with a term only of four months. Since then the courses have been lengthened to three years, the terms having grown to seven, eight, and even nine months, and a few colleges have a course of four years.

In a paper read by Dr. Truman W. Brophy before Section I. of the International Dental Congress at St. Louis, he said, "The course adopted in St. Louis, in July last, is of three years, each year to consist of thirty weeks, of six days in each week, exclusive of holidays. In thirty weeks of six days each we have one hundred and eighty days. This multiplied by three gives us five hundred and forty teaching days. The advantage in time which the *seven* months' course of four years has over a six months' course of four years is ninety-six teaching days. The advantage of the four years' course of seven months over the three years' course of thirty teaching weeks of six days in each week, is only forty-four teaching days. The advantage of the three years' course of thirty teaching weeks of six days per week over the four years' course of six months each, is forty-two teaching days." This, gentlemen, seems too trifling to make a great difference in learning, with any willing student of diligence and application.

I agree with Dr. Godon when he said, in the discussion of Dr. Brophy's paper, that we should not consider a four years' course of seven months or six months in its comparative value to the one of three years and seven months; but should only consider a four years' course of nine months. This ideal time will come, but not until all States and schools make their preliminary requirements alike.

Education is like freedom,—only a certain amount of it is good for man, and when beyond that limitation, slavery and over-education commence. The conception of education is most varied and elastic. There is a minimum of education which man and beast have in common. Ditte, in his history of education, says, "The sum of all the active services to be rendered by matured individuals to others not yet enabled to self-preservation, in order to secure their existence until they are able to sustain the same by their own powers;" and a maximum being "the sum of intentional and planned influences of developed mankind on the development of others not yet or less developed than their educators." Nature attributes the former to parents assisted in mankind by society. The latter is divided between parents and school,—its centre of gravity lying in the school, the only one of interest to us at present.

The meaning of school education is twofold: (1) the development of humanity to the characteristic of mankind; (2) instruction and training in arts and sciences, necessary for performing a part of the work on which each being depends for its subsistence, its happiness, comfort, and progress. The former begins with our birth and ends with death. Life remains the most permanent teacher, taking all of us to task, either sooner or later, for any carelessness in learning its lessons. The second is the task of the professional school. Proper training must prepare us for both this and the school of life.

The vast difference between the savage and our refined artists, poets, and scientists, lies in the difference of impressions made by the same objects on different minds by means of the very same sensual organs. The school of man must open our eyes for the evolution of nature and mankind; it must teach us to analyze and detail our sensual impressions; it must strengthen our power of observation, concentration, logical conclusions and expression; it must cultivate a sound mind in a sound body.

All skill and knowledge the school imparts to us must have this end in view. Our public school must, in the first place, be an elementary school of preparation for the higher school of life, enabling us to understand its lessons and profit by them.

The history of education tells us that the neglect of early training of the senses and physical and mental faculties was connected with a low degree of culture and civilization in knowledge, science, and art, and also in morals; while the gradual rising of education in this line was accompanied by rapid progress in all.

In Germany, a country of the highest standing in science and learning in modern time, they had, almost to the sixteenth century, no permanently established schools for the instruction of the people at large but convent and cathedral schools, considering the education for priesthood the highest aim for any institute of learning. Virgil was, for them, a magician, and the reader of his works was in danger of being prosecuted for sorcery by the church authorities. The clergy, educated in such schools, incarcerated Galileo for teaching the motion of the earth around the sun, and forbade the study of anatomy and medicine.

Latin grammar, dialectics and rhetoric, music, geometry, arithmetic, and astronomy, or rather astrology, constituted the so-called higher education. The low standard of the university in the middle ages is strikingly characterized by the fact that, even in the sixteenth century, professors of the University of Wittenberg admonished their students, as Raumer tells us in his "History of Pedagogy," not to be discouraged in the study of arithmetic by the difficulty of this discipline, as its first elements were comparatively easy, and although *multiplication* and *division* required application, they might be understood by attentive scholars without great trouble. What would we think of a university the students of which must be taught the four elementary principles of addition, subtraction, multiplication, and division by a professor who considers the two latter of great difficulty, requiring close application?

In this sixteenth century, students entering a university had no previous instruction in geography, history, mathematics, etc. Physics and natural history were unknown to them. They knew nothing but Latin and Greek, which they had not learned in order to study the wisdom of the classics or ancient history, but merely to be able to imitate their style of expression. All preliminary education of those times had no other end in view but the cultivation of the faculty of expression in word and script, training its scholars in Latin, the universal language of the educated classes in the middle ages. All learning was memorizing of words and their connection in phrases,—it gave mere forms without substance. Such questions as, "How old was the angel Gabriel when he brought the message to Mary?" "What language is spoken by the angels?" "How could Christ have accomplished the work of salvation if he had come into the world as a pumpkin?" are a few cited by Raumer in his "History of Pedagogy."

Only a few isolated men obtained a higher standing; they became aware of the truth that all science is based on individual observation of things and facts, which requires training and cultivation in the early stages of life. The educational reformers who have emerged from the darkness of the middle ages, men like Luther, Bacon, Locke, and others, have opened our eyes to the fact that popular intelligence is the source of all human progress, and that it can and must be fostered by proper education in childhood.

Only by a very gradual progress school education was reached. It has become the task of the commonwealth (1) to develop in its children the faculties and endowments proper for the real type of humanity; (2) to equip them by proper training with the knowledge and skill necessary for the entrance in a professional school. Education of the first kind it must make to a large degree obligatory; the latter must be optional. Languages, geography, history, arithmetic, the elements of physiology, natural history, and physics have become the general standard of a common-school education. Modern education has added to these instructions both music and drawing, having become aware of the importance of æsthetics for the development of true humanity which is based on both intellect and feeling.

Manual training has also been added, the educational experiments of Pestalozzi and Froebel having shown the immense educational value of an early training in this line by simply using the child's natural impulses to play and imitate. In a paper read at the National Meeting at Niagara Falls, in 1899, I gathered statistics to show the influence of manual training on general learning. The more complete this elementary department of education fulfils its task, the better will be the work of the later preparatory school, college, or university.

A good deal of the instruction now crowded into the high school might be distributed to the lower grades. The study of history, the elements of natural history, and Latin begin in Germany in the lower gymnasium at the age of eight to nine. Drawing is added in the next year; French, in my own school days; now English; the elements of mathematics in the following grade; Greek, the elements of physics, chemistry, and mineralogy before the pupil has reached his twelfth year. Most of these branches are still exclusively crowded into our high schools, thereby making the work of pupils and teachers burdensome, and not always allowing sufficient time for thoroughness and diversification.

When we consider that there is only a three years' course in most high schools, we shall understand that there can be but little time left for special preparation of any kind, and that its extension to four years is therefore an urgent necessity. Dr. Muensterberg, in his "American Traits," says, "At fifteen I was in the Untersekunda, and there is not the slightest doubt that, at that stage, all my classmates were prepared to pass the entrance examinations for Harvard College."

Dr. G. B. Snow, in an essay on dental education, tells us that the proposal was recently made by the dean of one of the best and most successful dental colleges in the country, that the first year be devoted to a line of subjects which are, or should be, taught in high schools, that the student might have a proper understanding of them before proceeding to his dental course. This would mean a correction of the insufficiencies of our high schools, and really implies that the dental course would be long enough if only the preliminary training were quantitatively and qualitatively sufficient and correct.

With some of the subjects now taught at the high schools referred to the lower grades, and with all States demanding a four years' course of high school training, we would be in a better position to classify the students according to the future study they intend to pursue.

Numerous preliminary studies are less essential to the doctor and the dentist than they are to the engineer. Other preliminary studies are only of value to the future pedagogue and the theologian, and would not deprive the medical or dental student of any necessary acquisition or culture for his vocation. Probably no preliminary studies require more, let me say, specialization, than those applied later on to the surgeon and dentist.

Broad culture can only be obtained by the mastering of manifold studies early in life; but, owing to the enormous extension of all individual sciences, we are apt to be taught far too much, which we cannot assimilate to proper advantage later on. If it is necessary to specialize and sub-specialize every profession, it will become equally necessary to specialize in the preliminary education of the future professional man.

Nothing is more essential than to bring the mind to a properly and correctly trained condition, where it will grasp with ease the scientific and technical teachings of the dental curriculum.

I consider the question of extending the course of dental teaching less important than the improved and extended preliminary teaching. Harvard has answered this question in a very emphatic manner.

Next January the requirements of the State of New York, as to preliminary education for the dental student, are to be raised to four years of high school education, or forty-eight counts. This is a decided step in advance, and the only reason the dental colleges of the State of New York have voted against a four years' course at the present time. If all other States had required similar qualifications for entrance into college, the action of the New York Colleges would have been more open to criticism. The great deficiency of this law remains, the fact that these counts may be obtained by passing examinations in any of some seventy-six branches, many of which are irrelevant to the future dentist.

It is certainly a wonderful progress in comparison to the time when the student was asked what language the angels spoke, but it is also a wonderful progress in comparison to the time when the student came directly from the plough and was told that "Levator labiæ superioris alæque nasi" was one of the many anatomical terms he had to remember for some of the smallest muscles of the body.

The higher the level on which the professional specializing begins, the more effective it is, and I believe also that the earlier we begin, the more effective it becomes. As Professor Muensterberg says, "In the kindergarten I should show my little lawyer two cakes, and explain to him that one is his cake and the other is not,—social information which does not lie in the line of my little naturalist; and I should tell the other little fellow that one cake has plums, and the other has not,—scientific instruction which is without value for the future lawyer."

Many students are destitute of the faculties most needed for the dentist because early training in childhood has been neglected. Manual training, especially, has not begun in the kindergarten and continued throughout the common school in concentric circles. I firmly believe that the principles and aims of general and special preparatory education, and their wants and shortcomings in their present state, form the most important subject for consideration in connection with dental education.

Professor Butler, in an essay on the "Function of the Secondary School," says, "One of the best known academies in the United States requires for admission only some knowledge of common school arithmetic, writing, spelling, and the elements of English grammar, and that the average age of pupils on entering is sixteen and one-half years. At this age the French and German boy is reading Cicero, Virgil, and Horace, Sophocles and Plato, Shakespeare and Tennyson, as well as studying general history, solid geometry, and chemistry. It is very evident that at this point there is a tremendous waste in our educational system. It must be remedied speedily, if our higher education is not to be discredited altogether." When this is happily solved, the proper length of the dental course can easily be ascertained and established.

A great deal of that preliminary education must consist of what I have written in a paper on "Manual and Artistic Training," *Dental Cosmos*, 1899, p. 1099: "Together with the training of the mind must be that of the hand and eyes, if we are not destined to that fatalism in operative dentistry that is beginning to make itself so brutally felt. The intellectual stimulus of constructive art is more vigorous than that of literature; we are continually stimulated by the presence of the object and the operations to be performed. The perceptions become clearer, more positive, more precise. It is a great mental discipline; yes, I go so far as to say it is a great moral discipline. It leads to more exact action; it leads to greater honesty, for honesty is but the moral expression of exactness."

